# Limits to truth-telling: Neurologists’ communication in conversion disorder

**DOI:** 10.1016/j.pec.2009.05.021

**Published:** 2009-11

**Authors:** Richard Kanaan, David Armstrong, Simon Wessely

**Affiliations:** a King's College London, Department of Psychological Medicine, Institute of Psychiatry, UK; b King's College London, Department of General Practice, UK

**Keywords:** Conversion disorder, Factitious disorder, Malingering, Hysteria, Truth-telling, Deception, Neurology

## Abstract

**Objective:**

Neurologists face a dilemma when communicating with their conversion disorder patients – whether to be frank, and risk losing the patient's trust, or to disclose less, in the hope of building a therapeutic relationship. This study reports how neurologists in the UK described dealing with this dilemma in their practice.

**Methods:**

Practicing consultant neurologists from an NHS region were recruited by snowball sampling. Twenty-two of 35 consultants in the region were interviewed in depth, and the interviews qualitatively analysed.

**Results:**

The neurologists were reluctant to disclose conversion disorder as a differential diagnosis until they were certain. They were guided by the receptivity of their patients as to how psychological to make their eventual explanations, but they did not discuss their suspicions about feigning. They described their communications as much easier now than they had seen in training.

**Conclusion:**

Neurologists adapt their disclosure to their patients, which facilitates communication, but imposes some limits on truth-telling. In particular, it may sometimes result in a changed diagnosis.

**Practice implications:**

An optimum strategy for communicating diagnoses will need to balance ethical considerations with demonstrated therapeutic benefit.

## 1 Introduction

Communicating medical information has become increasingly recognised as a core medical skill and responsibility. Though cultural differences remain, there has been a large shift in the expectation of disclosure of even terminal diagnoses in the West from the 1960s onwards [1]. This reflects a re-ordering of the ethical obligations on doctors: the previous paternalist position – protecting the patient from devastating news – has been subordinated to respect for patient autonomy in the form of ‘truth-telling’ [2]. But difficulties with truth-telling remain, and nowhere more prominently than in the field of medically unexplained symptoms. There, the principle of respect for autonomy can be set against the principle of beneficence [3], since giving an unwelcome psychiatric diagnosis for a somatic complaint may threaten the therapeutic alliance, with an angry patient dropping out of the clinic and treatment [4].

The paradigm unexplained syndrome is hysteria, or ‘Conversion Disorder’ as it is now known. Patients with conversion present with symptoms, such as paralysis, which appear at first to be neurological in origin but which on closer examination do not fit with known anatomy, physiology or neuropathology. Since the time of Freud psychiatrists have tried to offer psychological explanations for these symptoms (which remain ‘medically unexplained’ however, because any such explanation is not ‘organic’ – i.e. not in terms of pathophysiology) and conversion is now considered a psychiatric disorder [5]. But conversion is still largely seen by neurologists, for whom it presents a “crisis” over what to say [6]. Clear explanations are desperately sought by patients [7], and are thought to be key to eventual recovery [8], but any explanation seems fraught with risk. The neurologists tend to endorse the view that the condition is psychiatric [6,9,10], but the psychiatric models may seem outdated, not having advanced since Freud [11], and may be equated with feigning by patients [12]. The neurologist may also harbour suspicions that the patient is at least in part pretending [6,10,13], a view they may be reluctant ever to discuss [14].

The neurologist dealing with the conversion patient is thus faced with a dilemma: should they tell the patient what they think, and risk an angry breakdown in their therapeutic relationship; or should they offer something more acceptable to the patient, avoiding discussion of psychiatry or deception, and so gradually win them over to effective treatment [15]? Though the problem has been raised, neurologists’ response to that dilemma has not been reported. In this paper we examine their response – what they say in their management interviews with conversion patients, the pressures they feel and the choices they make.

## 2 Methods

All practicing consultant neurologists in an NHS region were approached, initially by email (a ‘consultant’ is the British term for a doctor who has completed all specialist training). Further recruitment proceeded by snowball sampling within the region until thematic saturation was reached. After giving written informed consent, the neurologists underwent depth interviews by the lead author, at a time and place of the neurologist's choosing – usually either the neurologist's office or a designated interview room, but on two occasions in the office of the interviewer. The interviews lasted from 35 to 70 min. The subject was ‘conversion disorder’, and no definition was provided by the interviewer. The topic guide covered the neurologist's background, training, and current practice; it asked for examples of memorable or current patients considered to have conversion, before seeking their definitions, exclusions, models and diagnostic practices, including the diagnostic interview and subsequent communications. The guide provided a bare structure, however, and the interviewer adapted the questions to the subject and their previous answers. The topic guide was reviewed and revised after piloting on the first five subjects. Interviews were audio-recorded, transcribed and analysed using NVIVO 7 software. The lead author listened to the recordings and read the transcripts – data collection and analysis proceeding concurrently. Transcripts were coded by question, and, iteratively, by emerging themes, consistent with a grounded theory approach [16]. Themes were reviewed with the other authors on an ongoing basis. The study was approved by the local Research Ethics Committee.

## 3 Results

### 3.1 Sample characteristics and background

Twenty-two neurologists were interviewed, from a potential pool of 35 and three more agreed but no suitable time could be arranged within the study period. Those interviewed ranged from 39 to 63 years of age, with a median of 45. Seven were female, 15 were male. Seventeen were Caucasian, two were from the Middle East and three from the Indian subcontinent. They had been medically qualified for 14–39 years (median 20 years), receiving their medical training in the UK (15 of them), other developed countries (5), and the subcontinent (2). Five had worked as psychiatrists earlier in their training (for from 6 weeks to 3 years), and eight had worked as neurologists on a specialist neuropsychiatry service. Many had exposure to conversion disorder, or a condition they considered similar, prior to qualification – seven had recognised it in family members, one in a friend, and one in their younger self. In terms of current work, all were attached to the regional neuroscience centre, with most working in district general hospitals for the bulk of their practice. Three held academic appointments, though all with substantial clinical commitments. All were general neurologists, except for one who saw only headache, one who worked only with movement disorders and three who worked largely or exclusively with epilepsy. All but five said they saw conversion commonly in their work – up to 20% of their workload. One (who treated only headache) reported not seeing it at all, and wondered whether it still existed. One reported seeing it less commonly than earlier in their training, two felt that it was now uncommon, and one that it was rare to see it “in its severe form” (S06).

The neurologists described three ways in which they communicated with their patients about their diagnosis. Firstly, it was discussed as part of ‘the differential diagnosis’ (the range of diagnostic possibilities being considered before a final diagnosis has been reached), as investigations proceeded; secondly, it was discussed once a diagnosis was reached, in what we hereafter term ‘the management interview’; thirdly, and accompanying both of the above, it was detailed in the letters the neurologist would write to the referring doctor, but which were copied to the patient. As we shall see, different results were obtained for each type.

### 3.2 Discussing the differential diagnosis

Though neurologists reported being able predict a conversion diagnosis from early on in the process, there was near-total support for a thorough investigation in any case, raising the question of whether and when to mention conversion in the diagnostic process. On the one hand, introducing the idea of conversion at an early stage was seen as a way of making it easier to accept eventually, while on the other, neurologists were wary of the reaction of their patients while there remained any question over the diagnosis. So the neurologists described tailoring the discussion to the receptivity of their patient:

… I usually bring up the issue of stress if I suspect it's a factor quite early on and I really see how they respond to that. If they are absolutely adamant that's not an issue then I will go through the motion of just excluding the organic pathology… (S03)

I would generally try and [introduce the psychopathological] as early as possible, usually at the first time I meet them. But it depends, really, how certain I am. Clearly if I am not certain that it's physical then I would say, “Well we need to do some tests first.” (S02)

There were thus two reported reasons for not discussing conversion in the differential diagnosis – lack of receptivity, and lack of certainty – which together served to minimise the risk of unnecessary breakdown in the therapeutic relationship:

… I think if you say to a patient very early on “I think this is psychiatric” … and then it turns out you are wrong… you may have lost their trust and you may then find it more difficult to manage that problem… (S16)

Though the need for certainty also served to minimise the risk of what was seen as a peculiarly severe error in misdiagnosing the neurological as psychiatric:

…the opprobrium cast on doctors who misdiagnose organic symptoms as psychological is greater… from the patient, from the relatives; your reputation, your colleagues… (S22)

…trying to tell someone there isn’t a physical cause when there has been is quite hard …because we view psychological illness differently to physical illness. If I told somebody that they had a stroke and actually they had [multiple sclerosis] … I wouldn’t feel that bad about it… (S21)

Consequently, some neurologists described a tendency to seek out any neurological explanation, no matter how unlikely, even when that ‘organic’ component might be only a small part of the problem:

…my worry is obviously, as a neurologist and a clinician, not to label patients with it who have a subtle underlying abnormality that is elaborated… (S17)

That there might be some minor organicity underneath - that's why I investigate so thoroughly. (S22)

So the discussion of the differential diagnosis was recognised as desirable, but in practice limited by the receptivity of the patient, and by the reluctance to raise the issue while there was any doubt. This same ‘bespoke’ tailoring of the psychiatric diagnosis was described in the eventual management interview.

### 3.3 The management interview

The neurologists described how once they were sufficiently sure of the diagnosis they would have the ‘management interview’ with the patient to give their opinion. These would vary with how receptive the patient was to psychological explanations:

…it depends on the patient. Some patients, maybe you could explain to them that there is a non-organic basis… but the majority of patients don’t like to be told that… (S07)

It depends on how I read them, and it varies from the absolutely frank… the worst way …is kind of shrugging my shoulders and saying “crikey I don’t know what's wrong with you…” (S17)

They detailed a wide range of models or metaphors, and would also tailor these to their patients’ responses:

…you can use analogies of the sort of ‘mind forgetting how to send the messages down’, and that ‘there's some inhibition of the areas of the brain involved with initiating movement’ …we sort of get a feel from the patient about the type of language that they comfortable about using and the type of …explanations that they would find palatable. (S06)

But the approach also varied by neurologist, with some prepared to go far in exploring possible psychological explanations, while others adopted a ‘merely unexplained’ model – that they simply did not have an explanation. So, though most reported offering some kind of a psychological model:

I would say to them that sometimes we see this sort of problem due to psychological issues which are often subconscious, you are not aware that you are doing them… (S16)

I might say that it was a bit like a hypnotic trance, I might say that the brain can alter lots of our conscious world unconsciously. Sometimes I use the example of soldiers in the battlefield not feeling pain as an example of how the brain can subconsciously modulate our sensory perception. (S02)

Others would avoid any mention of psychology or psychiatry:

…if I really thought it was a conversion …the first thing I would do is encourage lots of physiotherapy, lots of positive reinforcement… “you will get better” …in the hope that they may not get entrenched in that pattern… and sort of show them that they can get out of it. (S05)

Even if pressed for an explanation:

I’d just admit complete ignorance … ‘in neurology, if 2,000 people come in the door, most don’t have anything physical but they have very real complaints … every branch of medicine is full of these symptoms, and our one in my patch is what you’ve got’. (S04)

I’ll say that… neurology is not able to give all the answers for everybody's symptoms. What as neurologists we can do …is test the system… but there comes a point … where we have to say “well actually, we haven’t got the answer…” (S14)

### 3.4 Putting it in writing

The consultants generally described their letters as following the information given in the interviews closely. And for most the letter was seen as helpful, even when the patient disagreed with the diagnosis:

I might say something along the lines of, “I introduced the possibility that the symptoms might have a psychological cause, Mr Jones didn’t feel that was likely, but nonetheless I think it's worth pursuing this” …I wouldn’t exclude it simply because the patient felt unhappy at that idea. (S01)

But for letters written in the early stages of the diagnostic process, there was a common note of caution about the declaring of information before the diagnosis was secure, a caution that had to be balanced with the need for honesty, or accountability: the neurologist had to be able to defend the letter, not offend or alarm the patient, while still telling the patient's general practitioner what they suspected. This was not seen as a problem affecting only this group – strikingly, some (S2, S12 and S17) compared mentioning conversion in the differential diagnosis to mentioning motor neuron disease (an incurable, degenerative disease, which is usually fatal within a few years [17]). The balance was reportedly often accomplished through the use of terms which are, in a sense, codes: words which have a manifest meaning, for the patient, and one that may have been spelled-out in the interview, but which has a fuller, or slightly different, meaning for the general practitioner:

I find that my editing time is endless… because, not just for the type of patient that you might be worried about but also, for example, if you’ve seen somebody and you don’t know yet they have motor neuron disease but it's part of your differential …you end up having to find another word that implies it but doesn’t say it; which they don’t understand but my medical colleagues understand … a lot of people use the term ‘functional’… (S12)

…like most people, I would use certain codes – ‘elaborated weakness’, ‘inconsistent’ and so on - and the patients will be able to read that … but because I’ve had an open conversation with them, I hope they know where I’m coming from. (S18)

What is rather striking about such descriptions above is that the consultant has apparently had a frank discussion with the patient, is careful not to exceed what the interview contained (one consultant described dictating their letters in front of their patients), yet still wants, or expects the receiving doctor to read something extra, something coded into their correspondence. This may partly reflect the earlier stage of the diagnostic process – as with motor-neurone disease, the consultant would not want the patient to worry until absolutely necessary (so, subject 18, above, was quite willing, once sure of the diagnosis, to give it to the patient: *…if it is a frank conversion disorder I*’*ll say*, “*I think this is a conversion disorder*”). But the stage of the diagnostic process does not entirely explain the coding since, for some, the coding was present at every stage:

I’ll often say “it was difficult to judge if there was weakness on the left side”… or “I was unable to comment about weakness on the left side”… which is my coded message … in a way I’m saying, “Look …I can’t find a problem with the wires… so we need to explore other issues”, but I’ll have talked that through anyway… (S14)

And for some there were obvious reasons to code, since the letter was understood as being ‘intercepted’ by the patient, rather than being for the patient:

I don’t use the letter as a communication between myself and the patient. It's more a copy of a medical document that goes between me and the GP … I try not to [omit things] because …I want to be able to trace my train of thought. (S19)

I’ve got to say what I need to say in the letter. It's primarily to communicate with myself next time I see them and to communicate with other physicians, and if I’m hampered in doing that it's to nobody's interest. (S17)

So, though we have described a general picture of the neurologists talking about the psychological, albeit with exceptions, and with caution, we are led to the question of what the neurologists may think, but not say to the patient.

### 3.5 What they do not say

Since our interviews also covered the neurologists’ beliefs about conversion, we can consider whether the neurologists’ reported beliefs matched what they told their patients. And, in general, they did – with some important provisos. The neurologists described conversion as a severe, neurologically unexplained disorder for which a psychological model might be available – albeit not usually from them. But many of them did not make a clear distinction between feigning and conversion [10]. And there were gaps between their beliefs and the discussion of both psychological models and feigning.

We have already reported how the neurologists described adapting their discussion to the receptivity of the patient. This resulted in weaker endorsement of the psychiatric in their discussion with the patient than with their interviewer (whom most will have known was a psychiatrist). But in some cases this meant that the psychological was apparently not mentioned at all, leaving the patient with the ‘merely unexplained’ or an unsubstantiated neurological diagnosis:

There's another approach which is kind of complicity. You know, I’ve got a few patients where I’ve never really confronted or gone into what I think … (S16)

Other reasons for omission were also reported, such as serving the greater good:

I was asked to review a lady with multiple sclerosis… she was the hero of this and that and worked terribly hard for people… I couldn’t find a neurological sign… and I just went, “It's very nice to see you. I think you’re doing really well and I don’t think there's any need for me to see you again.” (S10)

Or the therapeutic efficacy of avoidance:

… one makes sort of encouraging noises - a course of physio, come back and see us … and if they’ve done well then you just let sleeping dogs lie, I think. [Otherwise] it's …opening a can of worms really…: one, it may not be quite so serious; … two, there may not be a right way to address some of the issues. (S03)

Though in most this was described as occasional, there were some for whom it represented a more systematic avoidance of an uncomfortable scenario:

I try and avoid it as much as possible and I try and get other people to do it … so then obviously they can’t put questions… which I wouldn’t necessarily know how to answer… (S05)

In this context, the offering of second opinions was not infrequently invoked: though it acknowledged the lack of certainty, it could also serve to avoid making the diagnosis:

If it's very difficult I might … offer them a second opinion … I do that less and less … I began to realise I was actually copping out of just confronting the situation. (S17)

The neurologists were divided on the role of feigning, usually described as ‘malingering’. There were some who maintained a clear division between feigning and conversion, but equally many who saw the division as unclear, in a number of ways: some held that the two could not be clinically distinguished, some that the disorders were blurred together, and some who saw feigning as being ubiquitous and hence not an exclusion criterion [18]. None of the neurologists reported discussing feigning as part of a differential diagnosis, however.

I may be having some private thoughts about the possibility of factitious disorder, but …I don’t put that to patients. I don’t have… a good way of saying, “As well as being epilepsy and these other things, non-epileptic attack disorder, you could just be making this up.” (S01)

Even in those rare cases where neurologists reported being convinced that a patient was feigning it was very unlikely for them to discuss this with the patient. One neurologist described confrontation of a patient with Munchausen's syndrome, but other patients ‘caught’ in acts of feigning or conscious control of their symptoms were more likely to be allowed to ‘slink off’ and self-discharge. If discussed at all, feigning was so heavily coded as to be almost indecipherable:

…if I think …there might be a more conscious process of the whole thing taking place… I would put that in a very subtle manner … “these symptoms are very rarely seen to co-exist together; one would therefore assume that there might be some other underlying features which we may not have identified…” (S20)

But the neurologists reported they usually ignored it, because it was too much trouble:

…if I really thought they were putting it on … I would provide them with the reassurance that I haven’t found anything and then sort of write back to their GP and explain that rather than trying to put them through the system. (S16)

Or too unpleasant:

…subconscious behaviour has nice connotations and conscious behaviour has nasty connotations …it forces people therefore to use - because they want to be nice to their patient - to use inappropriate diagnostic labels …to talk about a “conversion” or “hysteria” when actually they’re malingering. (S22)

Or because they did not feel it was a medical issue:

It's not your job to make any sort of value judgement… (S10)

### 3.6 Pressures in the interview

Many of the reported reasons for the gaps between preferred and achieved communication have been alluded to above, but we shall expand on them here. They may be broadly divided into desire for therapeutic effect and wish to avoid confrontation, though these often coincided.

The neurologists expressed the view that delicacy was required to obtain an optimal therapeutic effect, to allow the diagnosis to unfold in a way that the patient could eventually accept, or to accept a treatment they may otherwise reject. And they described learning this both through their training and experience, but also particularly the older doctors through seeing their teachers’ difficulties with a more challenging approach:

I saw too many of my senior colleagues… losing contact with their patients by saying, “We can’t find anything wrong with you - goodbye” … it's a fundamental failure of the role of the physician. (S18)

…when I was training it used to be a stressful and difficult thing to do and now I don’t find it … there was a sort of, “it's real or it isn’t real”, and “you might be putting it on” and …that's what got the doctor and the patient annoyed. (S04)

On the other hand, the neurologists were frank about their more personal motivations, of the chastening experiences of mistakes, complaints and court cases, and, at a more mundane level, of the desire to have pleasant and respectful relationships. For though many patients responded positively, the relationship could be jeopardised by giving an unwelcome diagnosis to an unreceptive patient:

It's variable really. Some people are very acceptant of it …jumping up and down and very keen to see the psychiatrist…some people say thank you very much and go out the door and then don’t come back (S16)

It evokes various types of responses in people …some… get quite angry… “are you saying it's in my mind?” …another …response is …“so that means you don’t really know what's going on…” (S20)

…the two things, if I’m honest, are a dislike of conflict… and, I guess, deep down, the desire that when somebody leaves the room… they don’t hate me. (S18)

…it's so unusual for us to have a patient who aggressively disagrees… it's very unusual …that you’re not on the same side as them… (S06)

To that end, giving some patients a way out of their difficulties, whatever the neurologist might believe about their aetiology, could be both therapeutically effective, and also avoid conflict:

…if you suddenly come at them and sort of say “oh, stand up you fool” …I don’t think you’re going to really benefit that patient or that patient's family. Whereas if you can introduce the concept of being subconscious… then I think you keep the patient's trust on board and they are more likely to go ahead with you. (S16)

…sometimes you need to… to some extent play along, have a face-saving formula and get out of it together… (S12)

## 4 Discussion and conclusion

### 4.1 Discussion

Faced with an apparently stark dilemma – tell the truth and lose the patient, or dissemble and help them – the neurologists we interviewed described piloting a middle way. They described seeking a way of saying what they believed, more or less, while still helping the patient. If this were borne out it could be seen as a success for the efforts that have gone into communication training for clinicians. Tailoring the discussion, with an ear for the patient's view, is not only good communication, it is also good medical practice (see, for example, the General Medical Council's Guidelines [19]). And this group of neurologists described far less hostile and far more therapeutic patient relationships than they saw in their predecessors. However, there was also a cost to the approach the neurologists described, in that at least some neurologists, with some patients, were not saying quite what they believed. Most neurologists strove to give the patients an acceptable psychological model for their symptoms, but the pressure of adapting would often mean that the psychological was minimised, and would sometimes mean that the psychological and in almost every case that feigning were not mentioned at all.

In considering whether this matters we must ask what the neurologists are doing in their adaptation. In deferring the differential diagnosis, the neurologist is being classically paternalist. They defer the discussion until they are certain, sparing the patient (and themselves) an awkward, and hopefully unnecessary, period of stress. Concern that the neurologists may be motivated by the desire to avoid conflict can be largely set aside: avoiding conflict can itself be seen as therapeutic, or at least as coinciding with therapeutic goals, as a ‘double effect’ (as when giving a lethal dose of morphine serves the double effect of analgesia). And though it is perhaps surprising that a differential diagnosis of conversion would be thought as difficult to communicate as a terminal illness, the handling of such illness does provide a clear precedent for what the neurologists do here. As long as the patient is eventually given the results, it can be argued that no harm has been done [20]. The situation is consequently different in the management interview, since the diagnosis by that stage is as clear as it is ever likely to be. There, the question can be framed as ‘when does adapting the message become collusion?’. The distinction is likely to be a very fine one, but one key criterion could be – if it changes the diagnosis.

One difficulty with the field of unexplained neurology is that there are several competing diagnoses which neurologists find poorly demarcated from each other [10]. Nevertheless, if we reduce the differential diagnosis to three – the physical unexplained (there is a medical explanation, but we simply have not found it), the psychologically explained (conversion disorder), and wilfully explained (feigning) – then diagnosing one of these three may be understood to be excluding the other two. That is explicitly the case with the psychiatric criteria [21,22], and finds some support from neurologists [10]. In which case, by not pointing out the wilful or the psychological, the neurologist may be changing the diagnosis.

Though changing the diagnosis sounds serious in principle, however, such is the state of research in this field that we do not know whether it makes any difference in practice. The diagnoses are seen as woefully inadequate [23], so that some have called for the distinctions to be dropped [24], and, in any case, there is little evidence for effective treatment of any of the three [25,26]. So, should the neurologists do differently, and push their views harder on resisting patients? The neurologists described many situations when pressing frank views of feigning or of psychological explanations had led to complaints or therapeutic breakdown, suggesting that they had learned where to draw the line through hard-won experience. Yet there is evidence from primary care, at least, that much of the pressure for a somatic explanation does not come from the patient [27]. And just because patients resist a diagnosis does not mean they would prefer not to be told it. Patients in primary care were aware when their doctors ‘colluded’ with their views, and did not find it helpful [28]. They have expressed a clear desire for full disclosure of diagnoses as diverse as Alzheimer's and cancer [1]: whether that is also true for patients with unexplained neurology has yet to be determined, but a fair presumption would be that patients want to know what their doctors believe.

### 4.2 Conclusion

Communicating the diagnosis of conversion disorder was seen as potentially challenging by neurologists, who described adapting their communication to their patients’ receptivity to psychological thinking. This would sometimes emphasise therapeutics over the disclosure of the neurologist's opinion, minimising the role of psychology and ignoring any suspicions about feigning. In some cases it may have resulted in changed diagnoses.

### 4.3 Practice implications

Even where patients appear to disagree with a diagnosis, it should not be presumed that they do not want to know what their doctor thinks. The optimum strategy for communicating diagnoses of unexplained neurology has yet to be empirically determined, but it will need to balance ethical considerations with demonstrated therapeutic benefit.
